# Enlarging Red Blood Cell Distribution Width During Hospitalization Identifies a Very High-Risk Subset of Acutely Decompensated Heart Failure Patients and Adds Valuable Prognostic Information on Top of Hemoconcentration

**DOI:** 10.1097/MD.0000000000003307

**Published:** 2016-04-08

**Authors:** João Pedro Ferreira, Nicolas Girerd, Mattia Arrigo, Pedro Bettencourt Medeiros, Miguel Bento Ricardo, Tiago Almeida, Alexandre Rola, Heli Tolpannen, Said Laribi, Etienne Gayat, Alexandre Mebazaa, Christian Mueller, Faiez Zannad, Patrick Rossignol, Irene Aragão

**Affiliations:** From the INSERM, Centre d’Investigations Cliniques Plurithématique 1433, INSERMU1116, Université de Lorraine, CHRU de Nancy, F-CRIN INI-CRCT, Nancy, France (JPF, NG, FZ, PR); Department of Physiology and Cardiothoracic Surgery, Cardiovascular Research and Development Unit, Faculty of Medicine, University of Porto, Porto, Portugal (JPF); INSERM UMR-S 942, APHP Lariboisière University Hospital, F-CRIN INI-CRCT, Paris, France (MA, AR, HT, SL, EG, AM); Internal Medicine Department, Centro Hospitalar do Porto, Porto, Portugal (PBM, MBR, TA); Department of Anesthesiology and Critical Care Medicine, Saint Louis Lariboisière University Hospital, Assistance Publique – Hôpitaux de Paris; Université Paris Diderot, Paris, France (EG, AM); Cardiovascular Research Institute Basel (CRIB), University Hospital, Basel, Switzerland (CM); and Department of Cardiology, University Hospital, Basel, Switzerland; Intensive Care Unit, Centro Hospitalar do Porto, Porto, Portugal (IA).

## Abstract

Red blood cell distribution width (RDW) may serve as an integrative marker of pathological processes that portend worse prognosis in heart failure (HF). The prognostic value of RDW variation (ΔRDW) during hospitalization for acute heart failure (AHF) has yet to be studied.

We retrospectively analyzed 2 independent cohorts: Centro Hospitalar do Porto (derivation cohort) and Lariboisière hospital (validation cohort). In the derivation cohort a total of 170 patients (age 76.2 ± 10.3 years) were included and in the validation cohort 332 patients were included (age 76.4 ± 12.2 years). In the derivation cohort the primary composite outcome of HF admission and/or cardiovascular death occurred in 78 (45.9%) patients during the 180-day follow-up period.

Discharge RDW and ΔRDW were both increased when hemoglobin levels were lower; peripheral edema was also associated with increased discharge RDW (all *P* < 0.05). Discharge RDW value was significantly associated with adverse events: RDW > 15% at discharge was associated with a 2-fold increase in event rate, HR = 1.95 (1.05–3.62), *P* = 0.04, while a ΔRDW >0 also had a strong association with outcome, HR = 2.47 (1.35–4.51), *P* = 0.003. The addition of both discharge RDW > 15% and ΔRDW > 0 to hemoconcentration was associated with a significant improvement in the net reclassification index, NRI = 18.3 (4.3–43.7), *P* = 0.012. Overlapping results were found in the validation cohort.

As validated in 2 independent AHF cohorts, an in-hospital RDW enlargement and an elevated RDW at discharge are associated with increased rates of mid-term events. RDW variables improve the risk stratification of these patients on top of well-established prognostic markers.

## Abstract 2

Supplemental Digital Content is available in the text

## INTRODUCTION

Red blood cell distribution width (RDW) is a measure of size variability in the red blood cell population (anisocytosis). Its value is obtained by dividing the standard deviation of erythrocyte volume by the mean corpuscular volume and multiplied by 100 to express the result as a percentage.^1^ Disorders related to ineffective erythropoiesis or increased red blood cell destruction cause greater heterogeneity in erythrocyte size and consequently higher RDW.^2,3^ Hence, it has been advocated that RDW may serve as an integrative measure of nutritional deficiencies (e.g., iron and folate), bone marrow dysfunction, and/or systemic inflammation, all of which are associated with worse outcomes in heart failure (HF) patients.^2,4^ An elevated RDW has been associated with mortality in several settings, such as coronary artery disease, chronic HF, acute HF, stroke, pulmonary hypertension, peripheral artery disease, critically ill patients, and severe sepsis/septic shock.^4–11^ This finding is of major interest since RDW is inexpensive and widely available as part of the routine complete blood count.

Most studies have investigated the association of the RDW value with outcome using a single baseline measurement (in various cohorts); however, much less is known regarding the impact of the changes in RDW during hospitalization. In fact, RDW may be considered as a dynamic variable with rapid changes in acute disease states.^10^

Hemoconcentration (increase in hemoglobin and/or hematocrit during hospitalization) has also been studied has a possible target to assess decongestion in HF.^12–18^

To the best of our knowledge, no previous study has addressed the dynamic changes of RDW in acutely decompensated heart failure (ADHF). Furthermore, the relationship between evolving RDW and hemoconcentration during hospitalization has yet to be tested.

We hypothesize that an increased RDW from baseline to discharge can improve prognostic information in comparison to discharge RDW alone, while increased RDW without hemoconcentration may help to identify very high-risk ADHF patients.

## METHODS

### Studied Population, Emergency Room Description, and Oversight

We analyzed 2 independent cohorts:

1. Porto cohort (derivation cohort) – during a 3-year period (from January 2012 to December 2014), all patients with ADHF admitted to the emergency room (ER) of the tertiary university hospital *Centro Hospitalar do Porto* (CHP), Porto, Portugal, were retrospectively studied. This ER has moreover certain noteworthy particularities. The ER is situated within the Urgency Department under the supervision of the Intensive Care Unit. The ER is equipped with ventilators and invasive monitoring devices in order to receive unstable/severe patients. The patients described in this cohort were all admitted for ADHF/pulmonary edema with associated respiratory insufficiency (PaO_2_/FiO_2_ < 300)
2. Paris cohort (validation cohort) – during a 4-year period (from January 2011 to December 2014), all patients with dyspnea admitted to the urgency of the tertiary university hospital Lariboisière, Paris, France, were prospectively recorded in a uniform database, which was retrospectively used for this study purposes, in which we selected only those patients an ADHF diagnosis.

All authors designed the study. The 1st, 3rd, 4th, 5th, and 6th authors collected, recorded, and adjudicated the data. The 1st 2 authors performed the statistical analysis and wrote the 1st draft of the manuscript. All authors edited and approved the manuscript and assume full responsibility for the accuracy and completeness of the data and for the fidelity of this report to the study protocol.

### Criteria and Definitions

**FIGURE 1:**
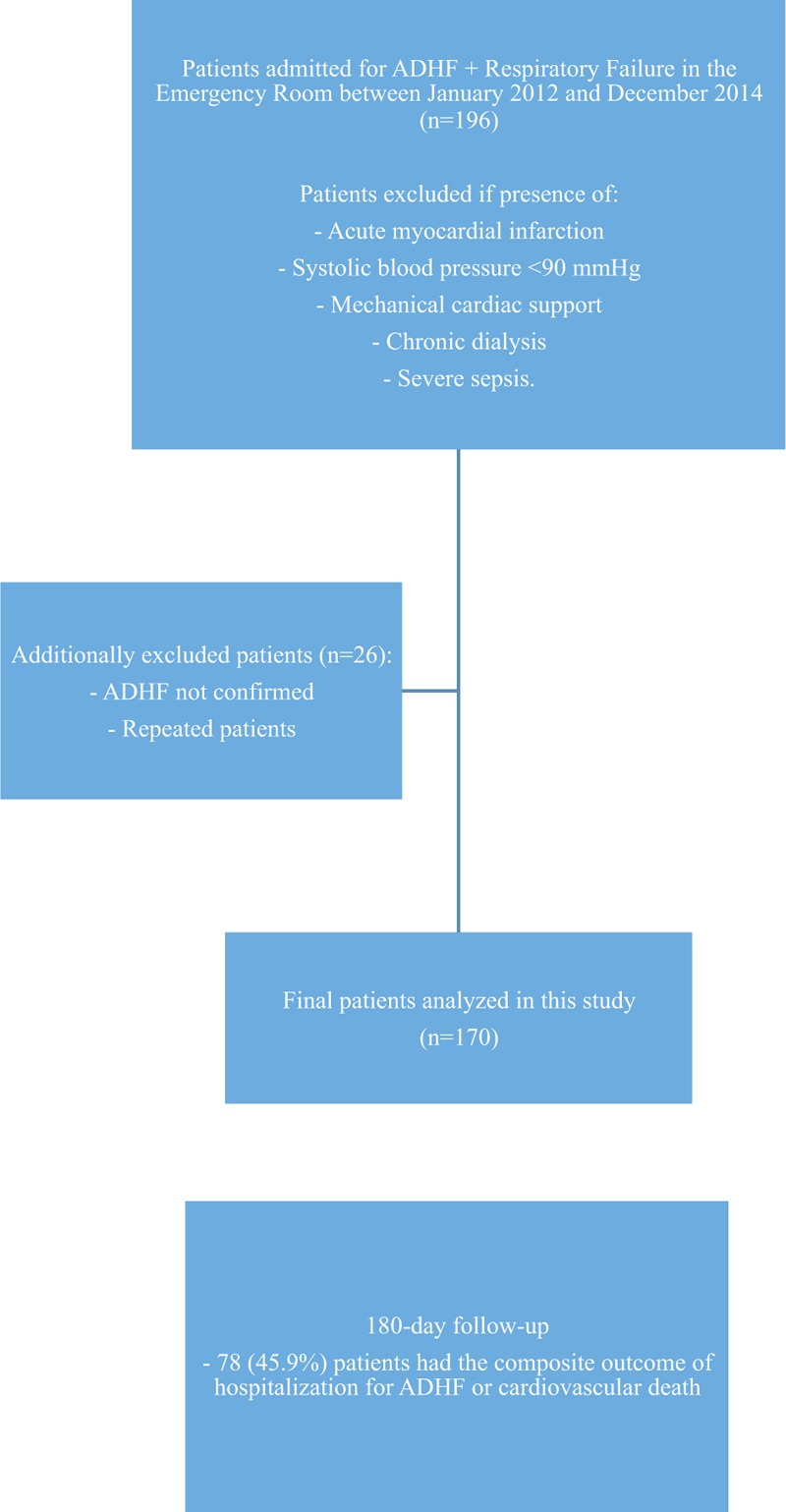
ADHF = acutely decompensated heart failure.

Patients with ADHF criteria were included. The diagnosis of ADHF was performed according to the European Society of Cardiology (ESC) criteria, defined as a rapid or gradual onset of signs and symptoms of worsening HF resulting in unplanned hospitalization (including new onset acute HF).^19,20^ Associated elevated natriuretic peptides (NPs) were used to adjudicate hospitalization whenever possible (83.5% of cases in Porto cohort and 96.4% of cases in Paris cohort). An echocardiographic study was performed on all patients during the index hospitalization. Left ventricular ejection fraction (LVEF) was measured by Simpson biplane method. Patients with acute myocardial infarction, systolic blood pressure <90 mmHg, mechanical cardiac support, chronic dialysis, and severe sepsis were excluded in order to mitigate inclusion bias and obtain a uniform dataset of severe ADHF patients – Figure 1.

Patient cases were recorded in a uniform database based on the information collected from the clinical records/reports. The study included cases from both community and referral hospitals. Underlying diseases, precipitating factors, clinical presentation, most recent echocardiography findings, and analytical results (including hemoglobin, hematocrit, RDW, electrolytes, plasma creatinine and urea, and NPs) were recorded at admission (i.e., first available data) and discharge (i.e., the last available data). Blood analyses were performed using the Advia 2120 Hematology Analyzer (Siemens Healthcare Diagnostics, Deerfield, IL) in CHP laboratory and Cell-Dyn Sapphire Analyser (ABBOTT Diagnosis) in Lariboisière laboratories. The RDW value is reported as a coefficient of variation (percentage) of red blood cell volume. The normal reference range for RDW in these hospital laboratories is 11.5% to 14.5% with intra- and interassay variation coefficients of 3.6% and 6.5%, respectively.

The patients and/or their families who had been lost from electronic registries were contacted in order to incorporate an accurate, unbiased prognostic information.

In Porto cohort we studied 2 end-points: a composite outcome of hospitalization for ADHF or cardiovascular mortality (CVM) – whichever occurred first; and all-cause mortality (ACM). In Paris cohort we studied ACM as primary end-point, since the diagnosis for subsequent hospitalizations was not adjudicated.

Hospital admission for ADHF was defined according to the most recent guidelines.^21^ The follow-up period was 180 days starting from the hospital admission date.

The study was carried out in accordance with the Declaration of Helsinki and approved by the Institutional Ethics Committee.

The study flowchart for Porto cohort is provided in Figure 1.

### Statistical Analysis

The results are expressed as mean ± standard deviation (SD) if the continuous variables had a normal distribution or as median (percentile_25–75_) if the distribution was skewed. Normality assumption was verified by visual discretion. Categorical variables are expressed as absolute numbers (n) and proportions (%).

The delta (Δ) RDW was calculated as the difference between the last and first values divided by the first (last − first/first × 100) to account only for the actual difference between the 2 values. ΔRDW ≤ 0 was considered as a decrease while ΔRDW > 0 was considered as an increase. The first and last RDW were also dichotomized by rounded the median value (15% in both cohorts). In Porto cohort, delta hemoglobin (Hb) was also calculated, with ΔHb > 0 considered as a hemoconcentration, for example, an increase in hemoglobin from admission to discharge, and ΔHb ≤ 0 considered as no hemoconcentration, that is, a decrease or no increase in hemoglobin from admission to discharge. In Paris cohort, ΔHb was not calculated because discharge Hb values were not available in the dataset.

Population characteristics were compared using the independent sample *t*-test for normally distributed continuous variables, the Mann–Whitney *U*-test for skewed variables, and Chi-square tests for categorical variables.

Linear regression analyses were performed to assess the linear association between RDW and determinants of clinical, biochemical, therapeutic, and cardiovascular parameters. Candidate variables were chosen based on their a priori likeliness of being associated with RDW after which a backward selection was performed. Linearity and goodness of fit were verified. Binary logistic regression analyses were also performed to assess the associations of medications with dichotomic RDW.

The primary outcome was a composite of hospitalization for ADHF or CVM in Porto cohort and ACM in Paris cohort. Univariable time-to-event comparisons were made using the log rank test and univariable Cox proportional hazards models. Survival was estimated with the Kaplan–Meier method. Cox proportional-hazards models were used to obtain unadjusted and covariate adjusted hazard ratios (HRs). Proportional hazards assumptions were verified, with covariates used for adjusted HRs chosen from demographic, clinical, and laboratory variables that had been previously found to be clinically relevant. All continuous variables included in the model were verified for linearity.

The increased discriminative value associated with the “net reclassification improvement” (NRI) was assessed at 180 days.^22,23^ This method assesses the ability of a new model to reclassify subjects with and without a clinical event during follow-up. The ability of the new model to reclassify is summarized by the NRI statistic. The continuous NRI method developed by Uno^23^ and implemented in the survIDINRI package of the R software (The R Foundation for Statistical Computing) was used. The continuous NRI method does not require a prior definition of strata risk, thus considering the change in the estimation prediction as a continuous variable. The integrated discrimination improvement (IDI) was also calculated and assesses the difference between the integrated sensitivity gain and the integrated specificity loss due to the addition of the studied estimator to the prognostic model.

Statistical analyses were performed using SPSS 23 software (IBM Corp. Released 2013. IBM SPSS Statistics for Windows, Version 23.0. Armonk, NY: IBM Corp.) and the R software (The R Foundation for Statistical Computing).

A *P* value < 0.05 was considered statistically significant (including for interaction).

## RESULTS

### Patient Characteristics

In Porto cohort a total of 170 patients were included. In this cohort the mean ± SD age was 76.2 ± 10.3 years. Half of the patients were male, most had a history of hypertension history (87.1%), diabetes mellitus was present in 52.9% of the patients, and ischemic heart disease was the most frequent underlying cause for HF (56.5%). The mean LVEF was 43.8 ± 11.1% – Table 1. The mean PaO_2_/FiO_2_ ratio was 165 ± 86. At admission, the vast majority of patients (98.8%) had signs of pulmonary congestion as assessed by interstitial pulmonary edema on chest X-ray, 54.7% had pleural effusion and 58.2% peripheral edema. No significant differences between ΔRDW groups were found with regard to clinical congestion markers at admission. However, at discharge, a trend for a higher proportion of patients with peripheral edema was found in the ΔRDW >0 group (17.6 vs 31.5, *P* = 0.054). Patients with ΔRDW >0 had lower sodium, albumin, hemoglobin, and hematocrit levels as well as higher N-terminal-pro brain natriuretic peptide (NT-pro BNP), both at admission and discharge (all *P* < 0.05). A trend toward lower levels of serum iron during hospitalization was also found in patients with ΔRDW >0 (54.9 ± 32.4 vs 43.5 ± 15.0, *P* = 0.082) – Table 1.

**TABLE 1:**
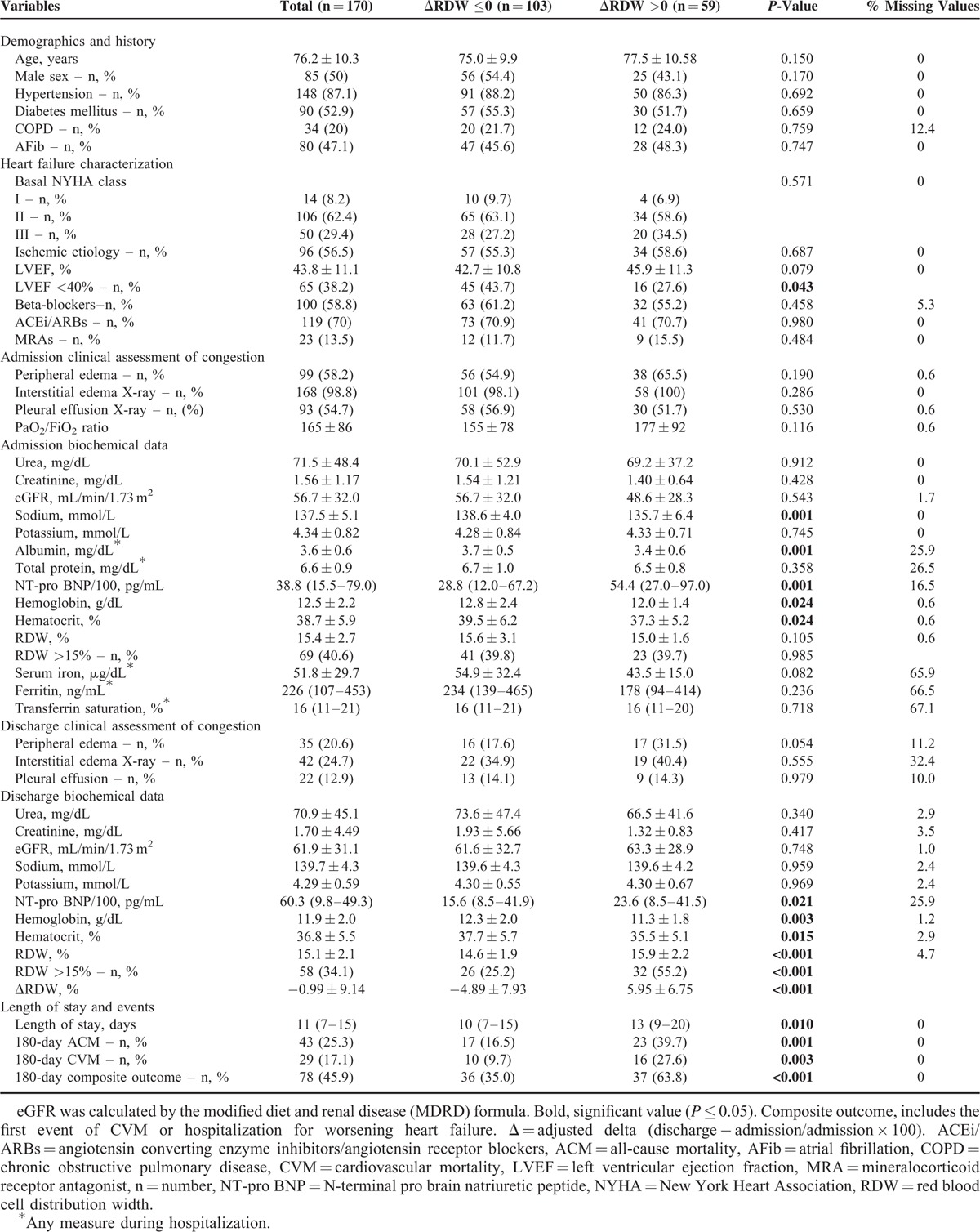
Characteristics of the Study Population Globally and According to RDW Changes

The composite outcome of hospital admission for worsening HF and/or CVM occurred in 78 (45.9%) patients, and 43 (25.3%) subjects died from any cause during the 180-day follow-up period – Table 1.

The baseline characteristics and events of Paris cohort are presented in the Supplementary Material Table 1.

### Factors Associated With RDW in Linear and Logistic Regression Analysis

In Porto cohort higher admission and discharge RDW levels were associated with lower levels of hemoglobin, hematocrit, and transferrin saturation levels (all *P* < 0.05). The presence of peripheral edema at discharge was also likely to be associated with increased RDW (*P* < 0.05). The ΔRDW was increased in association with a reduction in ΔHb (*P* < 0.05) – Table 2.

**TABLE 2:**
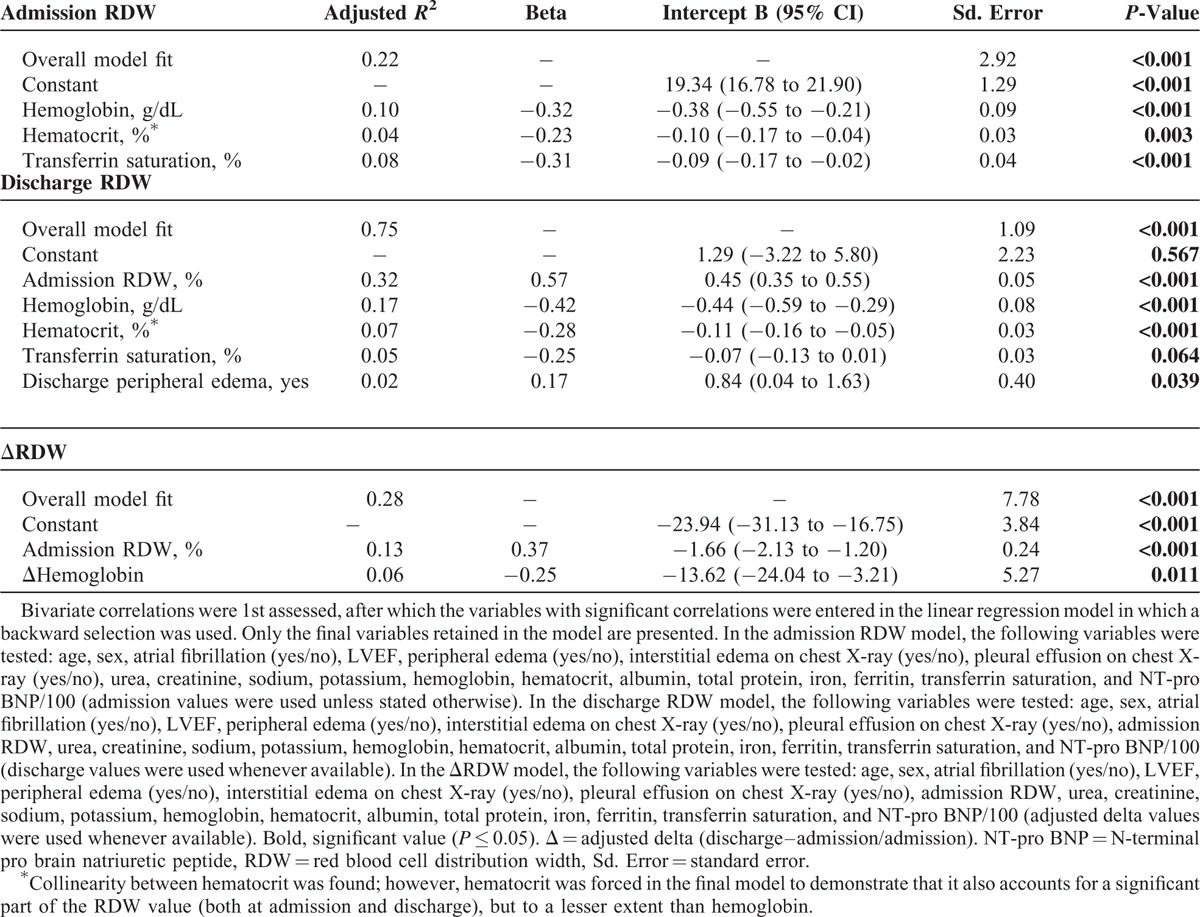
Multiple Linear Regression Analysis of RDW at Admission, Discharge, and ΔRDW

In Porto cohort, the odds of having admission RDW >15% were increased by the presence of atrial fibrillation, and the odds of having RDW >15% at discharge were increased by higher RDW value at admission (both *P* < 0.05). The odds of having ΔRDW >0 were significantly increased in patients with peripheral edema at discharge – Table 3.

**TABLE 3:**
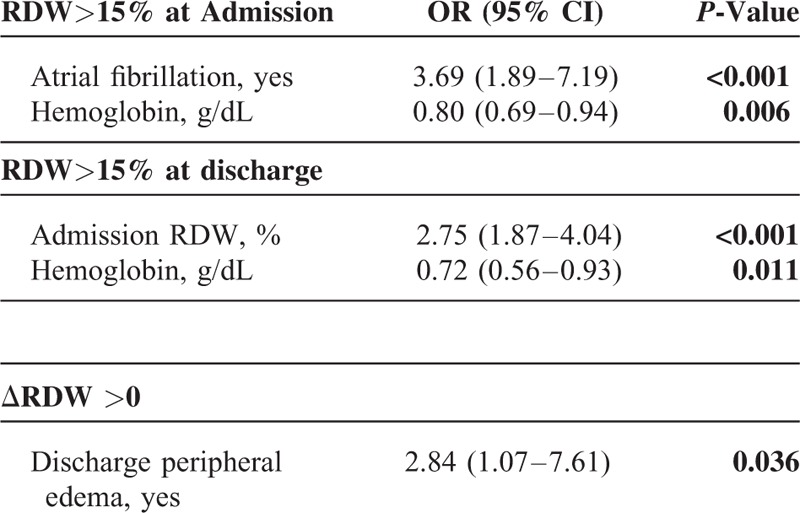
Multiple Logistic Regression Analysis of RDW (≤15 vs >15% at Admission and Discharge), ΔRDW (≤0 vs >0)

The factors associated with RDW values (both continuous and categorical) in Paris cohort are presented in the Supplementary Material Tables 2 and 3.

**FIGURE 2:**
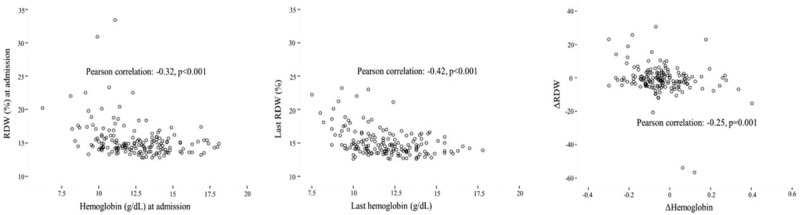
RDW = red blood cell distribution width, Δ = adjusted delta (discharge–admission/admission).

In Porto cohort admission, discharge and ΔRDW were negatively correlated with the corresponding Hb values (Pearson correlation = −0.32, −0.42, and −0.25, respectively, all *P* < 0.01) – Figure 2.

### Association of RDW With Outcome

**FIGURE 3:**
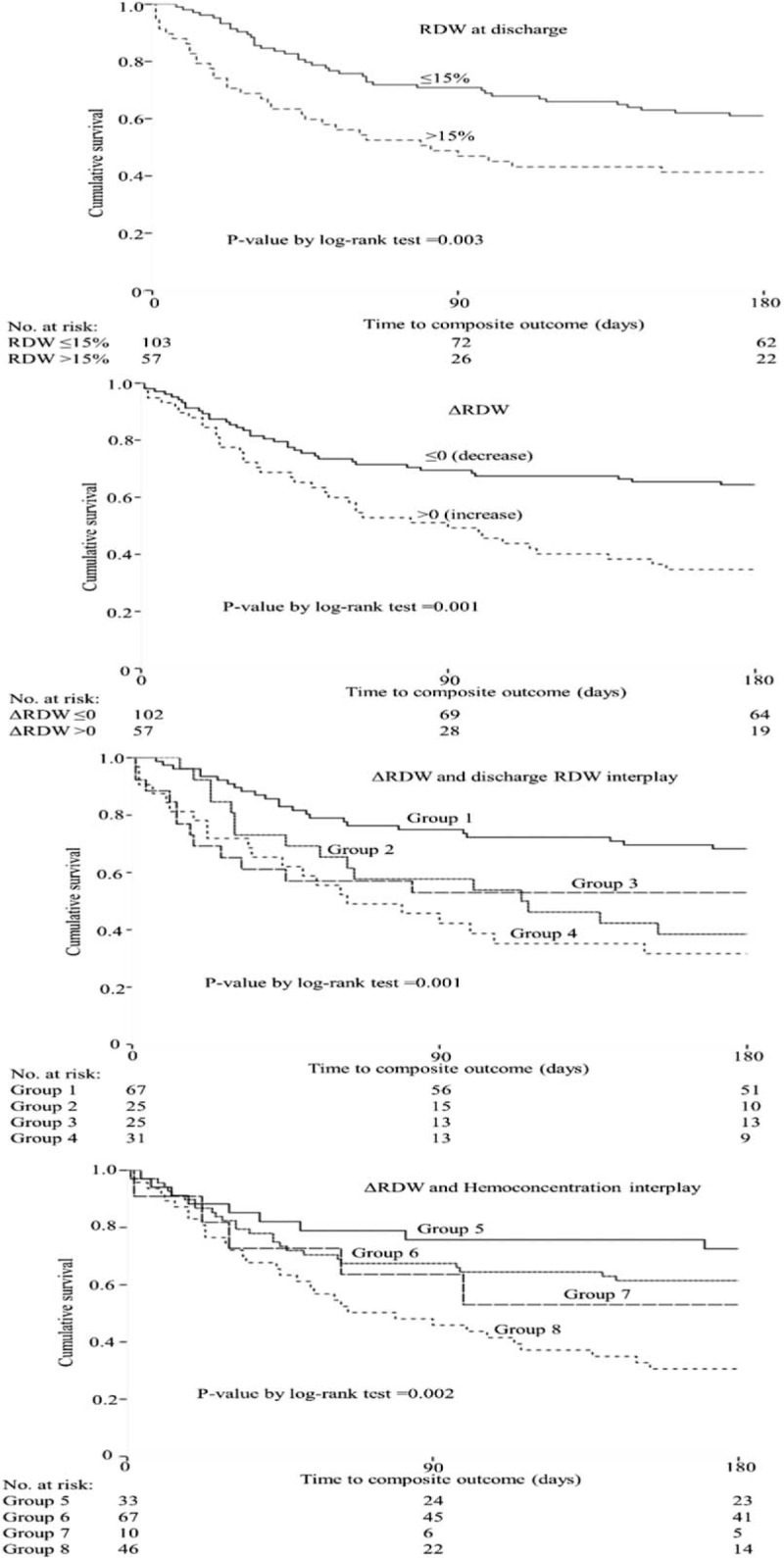
Group 1: RDW discharge ≤15% and ΔRDW ≤0; group 2: RDW discharge ≤15% and ΔRDW >0; group 3: RDW discharge >15 and ΔRDW ≤0; group 4: RDW discharge >15 and ΔRDW >0; group 5: ΔRDW ≤0 and ΔHemoglobin >0; group 6: ΔRDW ≤0 and Δhemoglobin ≤0; group 7: ΔRDW >0 and Δhemoglobin >0; and group 8: ΔRDW >0 and Δhemoglobin ≤0. RDW = red blood cell distribution width, Δ = adjusted delta (discharge–admission/admission).

In Porto cohort, the RDW value (continuous and categorical) at admission was not significantly associated with the composite outcome of heart failure hospitalization or cardiovascular death. In contrast, discharge RDW value (continuous and categorical >15%) was significantly associated with outcome. The last median RDW value >15% was independently associated with composite outcome, even after stepwise adjustment for potential confounders (sex, age, LVEF, atrial fibrillation, the presence of peripheral edema, NT-pro BNP level, sodium, urea, and Hb), whereas the last RDW as continuous variable did not remain significantly associated after adjustment for the confounding variables included in model 3 (the presence of peripheral edema, NT-pro BNP level, sodium, urea, and Hb) – Table 4 and Figure 3. Importantly, a ΔRDW >0 (i.e., an increase in the RDW value from admission to discharge) had a strong independent association with outcome (HR = 2.47 (1.35–4.51), *P* = 0.003, after adjustment for the variables included in model 3) – Table 4 and Figure 3. The strength of the association with the composite outcome was magnified when considering patients with both discharge RDW >15% and ΔRDW >0 (HR = 3.40 (1.63–7.08), *P* = 0.001, after adjustment for the variables included in model 3) – Table 4 and Figure 3. Considering ACM as outcome in Porto cohort, the associations of discharge RDW (continuous), discharge RDW >15%, ΔRDW >0, and discharge RDW >15% plus ΔRDW >0 together, were also positive and independently significant – Supplementary Material Table 6. Overlapping results were found in Paris cohort: the associations of discharge RDW (continuous), discharge RDW >15%, increasing ΔRDW, ΔRDW >0, and discharge RDW >15% plus ΔRDW >0 together, were also positive and independently associated with the outcome of ACM. Of notice, in patients with both discharge RDW >15% and ΔRDW >0 the association with outcome were particularly strong (HR = 3.82 (1.57–9.32), *P* = 0.003, after adjustment for the variables included in model 2) – Supplementary Material Table 4.

**TABLE 4:**
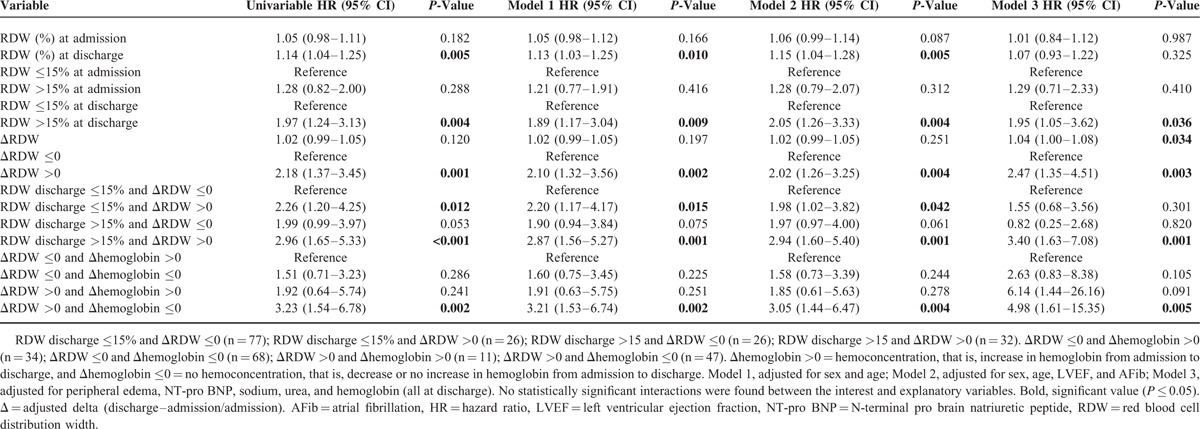
Univariable and Multivariable Cox Proportional Hazards Models for 180-day Composite Outcome According to RDW Values

### Association of ΔRDW and Hemoconcentration With Regard to Composite Outcome

In Porto cohort patients without hemoconcentration (defined by a decrease in Hb from admission to discharge or ΔHb ≤0) and ΔRDW >0, the association with adverse events was very strong (HR = 4.98 [1.61–15.35], *P* = 0.005, after adjustment for the variables included in model 3) – Table 4 and Figure 3. Of note, no significant interactions were found between the studied RDW values and several independent variables (including Hb). Hemoconcentration was not measured in Paris cohort due to unavailability of discharge Hb in the dataset.

### Net Reclassification Indices

**FIGURE 4:**
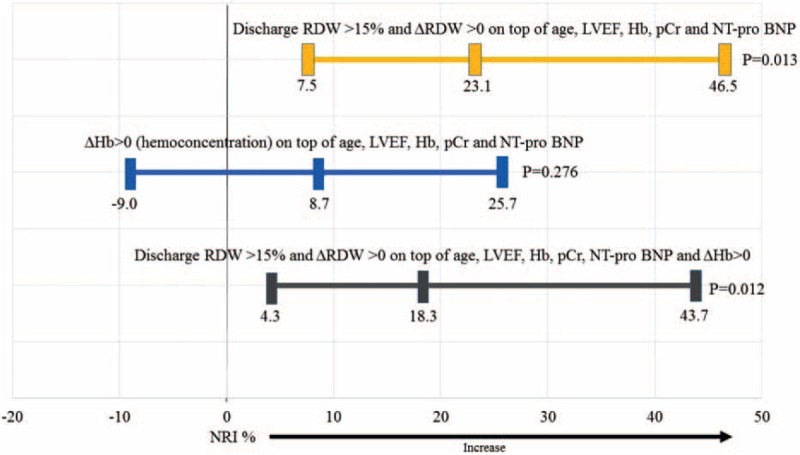
The prediction models include age in years (<77 vs ≥77), left ventricular ejection fraction in % (<45 vs ≥45), hemoglobin in g/dL (<12 vs ≥ 12), creatinine in mg/dL (<1.5 vs ≥1.5), NT-pro BNP in pg/mL (<3500 vs ≥ 3500) at admission, and Δhemoglobin >0 (hemoconcentration). ΔHemoglobin >0 = hemoconcentration, that is, increase in hemoglobin from admission to discharge, Δ = adjusted delta (discharge–admission/admission). Hb = hemoglobin, IDI = integrated discrimination improvement, LVEF = left ventricular ejection fraction, NRI = net reclassification improvement, NT-pro BNP = N-terminal pro brain natriuretic peptide, pCr = plasma creatinine, RDW = red blood cell distribution width.

In Porto cohort, the increased discriminative value associated with the addition of RDW and hemoconcentration on top of the covariates age, LVEF, Hb, creatinine, and NT-pro BNP at admission was evaluated in order to predict the 180-day primary composite outcome using NRI. The addition of the last RDW >15% and ΔRDW >0 in the survival model was associated with a significant improvement in reclassification (NRI = 23.1 [7.5–46.5], *P* = 0.013 and IDI = 8.2 [2.2–18.0], *P* < 0.001) – Table 5 and Figure 4. Of note, the addition of the last RDW >15% and ΔRDW >0 on top of the aforementioned variables plus hemoconcentration was also associated with a significant improvement in reclassification (NRI = 18.3 [4.3–43.7], *P* = 0.012 and IDI = 7.8 [1.2–17.1], *P* < 0.001) – Table 5 and Figure 4. Discharge RDW>15% and ΔRDW >0 alone also improved reclassification indices, whereas hemoconcentration alone did not – Table 5. In Porto cohort, the increased discriminative value associated with the addition of discharge RDW, RDW >15% and ΔRDW >0 on top of a survival model including the covariates age, LVEF, Hb, creatinine, and NT-pro BNP at admission also significantly improved the net reclassification for 180-day ACM prediction (NRI for discharge RDW continuous = 34.7 (−2.0 to 50.0), *P* = 0.073; NRI for discharge RDW >15% = 35.6 (3.6–55.1), *P* = 0.040; and NRI for ΔRDW >0 = 37.2 (6.0–55.4), *P* = 0.020) – Supplementary Material Table 7. Overlapping results were found in Paris cohort – Supplementary Material Table 5.

**TABLE 5:**
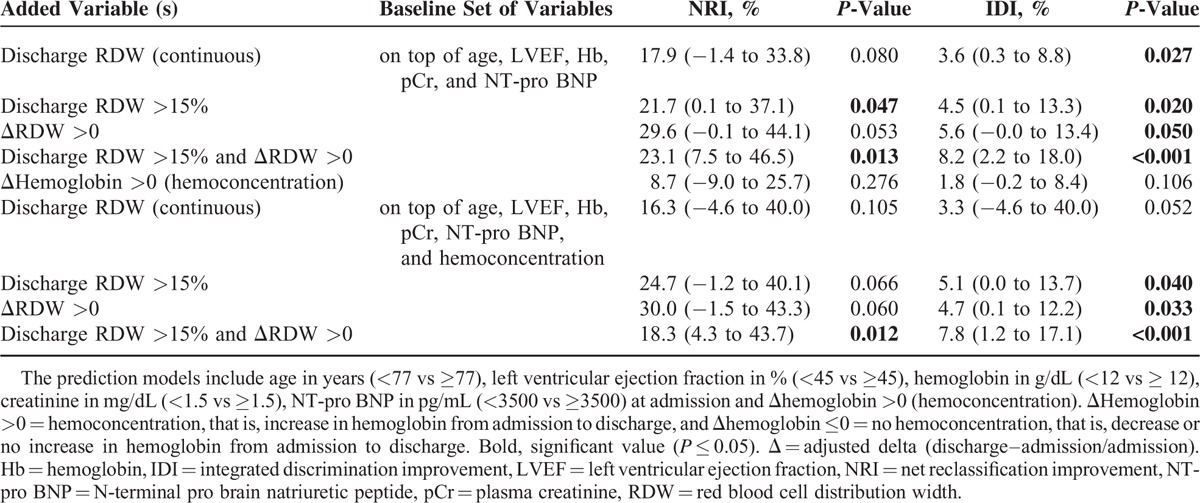
Net Reclassification Improvement and Integrated Discrimination Improvement for Predicting Composite Outcome at 180 days

## DISCUSSION

The present study shows that an enlarging RDW from admission to discharge and elevated discharge RDW values were independently associated with mid-term adverse events over and above hemoconcentration (as measured by a change in Hb during hospitalization). In addition, discharge RDW >15% and ΔRDW >0, either alone or combined (especially the latter), improved the prognostic model that included several well-established prognostic variables plus hemoconcentration. Except for hemoconcentration, these results were validated in 2 independent cohorts, and provide highly useful, simple, pragmatic, and costless prognostic information with the potential to be used in daily clinical practice.

### RDW as Prognostic Marker in Heart Failure and Its Incremental Prognostic Utility

The prognostic value of RDW in HF is well established in chronic HF. The CHARM (Candesartan in Heart Failure: Assessment of Reduction in Mortality and Morbidity) program included 2679 symptomatic chronic HF patients from North America in an analysis of 36 routine laboratory tests and their association with mortality. Higher RDW showed the greatest association with mortality and was among the most powerful overall predictors, with only age and cardiomegaly showing a better independent association with outcome. This finding was then replicated in the Duke Databank, in which higher RDW was strongly associated with ACM.^6^ In the ADHF setting the evidence is scarcer, nonetheless in 628 patients hospitalized for ADHF in Spain, a higher RDW at discharge (both continuous and categorical) was significantly associated with increased mortality independently of Hb value or anemia status. Additionally, RDW was also found to be associated with elevated troponin T, a marker of cardiomyocyte injury, and death in HF populations.^24^ These studies used a single RDW value as prognostic predictor. To the best of our knowledge, the present study is the first to show that a rising RDW during hospitalization is independently associated with adverse events (hospitalization and/or CVM) at 180 days. The predictive value of rising RDW was also present in patients with discharge RDW below the median of 15%. Of particular note, when rising RDW and discharge RDW >15% were added to a prognostic model including well-established HF prognostic markers (age, LVEF, Hb, creatinine, NT-pro BNP, and hemoconcentration), the capacity of the model to reclassify patients with and without event was significantly improved. The incremental prognostic utility of this finding merits serious consideration given its simplicity, its readily availability to all clinicians, without additional discomfort to the patients, and without cost escalation. This finding is of utmost importance since current HF risk prediction models do not take into account how individual patient assessments occur in incremental steps and, furthermore, each additional diagnostic assessment may add additional costs, complexity, and potential morbidity.^25^

### RDW and Hemoconcentration

Ineffective decongestion is responsible for many (≈35%) early readmissions,^26^ with a high burden both for the patient and the health-care system.^27^ The lack of available data to help clinicians in decongestion strategies is worrisome and requires further investigation.^28,29^ More recently, hemoconcentration has emerged as a possible target to assess decongestion in HF,^12–18^ particularly when assessed both at admission and discharge, since early improvements in congestion that are not sustained through hospital stay are not likely to be associated with improved outcomes.^30^ The difference between admission and discharge Hb is likely to be a good candidate to assess hemoconcentration.^29^ Moreover, our study showed that RDW is inversely correlated with Hb and adds prognostic information in addition to hemoconcentration, again with simple routine blood count values. It is thus likely that RDW and Hb evolving in opposite directions (increasing RDW and decreasing Hb) during hospital stay portends a worse prognosis.

### Potential Mechanisms

A rising RDW implies a reduction in structurally normal hemoglobin molecules.^3^ In our series, patients with ΔRDW >0 were more likely to have more peripheral edema, lower sodium, albumin, Hb and hematocrit levels, and higher NT-pro BNP at discharge. A trend toward lower levels of serum iron during hospitalization was also found in patients with ΔRDW >0. All of these factors are associated with worse prognosis in HF.^31,32^ The underlying mechanism to these associations has yet to be elucidated, although probably reflects a higher disease severity incorporating several pathophysiological processes such as nutritional deficiencies, inflammatory state, and renal dysfunction.^6,33^ In our study, RDW values are likely to be partially explained by hemoglobin/hematocrit (both admission and discharge RDW), transferrin saturation (discharge RDW), and by the presence of peripheral edema (discharge RDW). Nevertheless, a large proportion of variability in RDW cannot be explained by the several parameters tested herein, thus reinforcing the possibility that RDW serves as an integrative measure of several pathophysiological mechanisms such as anemia, nutritional deficiencies, and possibly congestion. Recent data have demonstrated that patients whose erythrocyte indices are evolving toward an iron deficient picture (i.e., rising RDW and falling mean corpuscular volume) have a higher risk of mortality, independently of their anemia status.^34^ Nonetheless, these data do not provide a clear basis for the prognostic implication of a rising RDW during such a short period of time (median length of stay in our dataset = 11 days), such that any potential conclusive explanation at this point is merely speculative.

### Clinical Implications

Our data provide useful clinical information derived from routine blood count without additional discomfort to the patient or cost increase. The present findings show that an enlargement in RDW from admission to discharge is associated with mid-term adverse events, even more so if the patients exhibit RDW >15% at discharge or no hemoconcentration (defined by Hb at discharge – Hb at admission), thereby adding further prognostic value to well established prognostic markers independently of hemoconcentration. As a result, this simple measure should help to identify patients likely to benefit from individualized strategies, thereby enabling a closer follow-up and/or tailored therapeutic strategies. This additional information particularly relevant in patients with ADHF renders serial hemograms even more useful during and possibly after ADHF hospitalization, with both hemoconcentration and RDW changes being assessed in routine clinical practice hemograms.

### Limitations

Several limitations should be acknowledged in this study. First, this is a 2-center, retrospective study (information was prospectively recorded in the validation cohort) with potential bias with regard to patient selection and information recording, although the present data were monitored in both cohorts and are consistent with previous studies in other fields as referenced in the discussion. Thus, our findings are likely to be generalizable. RDW was necessarily prospectively measured within the routine blood count (low risk of measurement bias – at least as low as in routine practice), was available in all but few patients (low risk of selection bias), and the used end-points were objective and collected in a standardized fashion (low risk of measurement bias). Our study thus avoided most pitfalls of historical cohorts.^35^ In addition, patient treatment was not tailored according to RDW values which further decreased the risk of bias. As a consequence, the results here presented are likely to reflect the prognostic value of RDW in daily clinical practice. Second, the use of erythropoietin was not investigated, and much of the data regarding iron parameters, folate or vitamin B12, are lacking or absent (as in Paris cohort). These values could provide further insight into the variation in RDW as well as in results interpretation. Third, these cohorts do not overlap the same patient-population, since Paris cohort subjects were likely to represent a less severe ADHF setting, as those were patients admitted to the general urgency, they did not necessarily have respiratory insufficiency, and had a much lower 180-day mortality rate. Nonetheless, Paris RDW results were also consistent with Porto results, suggesting that these findings can be generalizable to less severe ADHF populations. Fourth, we did not have discharge Hb in Paris cohort, hence hemoconcentration was not determined in this population. Fifth, discharge NP values could provide more accurate prognostic information; however, we used admission NPs in the net reclassification models due to the high percentage of missing discharge values. Last, the low number of patients in the 4 group categories reduces the precision of the estimated associations.

## CONCLUSION

As validated in 2 independent ADHF cohorts, an enlarging RDW during hospitalization for ADHF is associated with adverse outcomes. The prognostic value of elevated discharge RDW and rising RDW adds significant information (as assessed by net reclassification methods) on top of well-established prognostic variables (including hemoconcentration). These inexpensive and easily available biomarkers could help refine mid-term risk-stratification of patients admitted for ADHF.

## Supplementary Material

Supplemental Digital Content
