# Enlarging red blood cell distribution width during hospitalization identifies a very high-risk subset of acutely decompensated heart failure patients and adds valuable prognostic information on top of hemoconcentration: Erratum

**DOI:** 10.1097/01.md.0000503451.68473.25

**Published:** 2016-10-07

In the article “Enlarging red blood cell distribution width during hospitalization identifies a very high-risk subset of acutely decompensated heart failure patients and adds valuable prognostic information on top of hemoconcentration”,^[1]^ which appeared in Volume 95, Issue 14 of *Medicine*, Dr. Tolppanen's name was originally spelled incorrectly. The article has since been corrected online.
