# Supplementary material for: Enlarging Red Blood Cell Distribution Width During Hospitalization Identifies a Very High-Risk Subset of Acutely Decompensated Heart Failure Patients and Adds Valuable Prognostic Information on Top of Hemoconcentration
Source: Medicine (Baltimore). 2016 Apr 8;95(14):e3307. doi: 10.1097/MD.0000000000003307 (PMC4998821; doi:10.1097/MD.0000000000003307)
Supplement: Supplemental Digital Content [file medi-95-e3307-s001.doc]

Supplemental Table 1. Characteristics of the study population globally and according to RDW changes (Paris cohort)

| **Variables** | **Total** (n=332) | **ΔRDW ≤0** (n=209) | **ΔRDW >0** (n=123) | **P-value** | **% missing values** |
| --- | --- | --- | --- | --- | --- |
| **Demographics and history** | | | | | |
| Age (years) | 76.4 ± 12.2 | 76.0 ± 12.1 | 76.9 ± 12.4 | 0.519 | 0 |
| Male sex – n. (%) | 184 (55.4) | 117 (56.0) | 67 (54.5) | 0.789 | 0 |
| Hypertension – n. (%) | 234 (70.5) | 148 (70.8) | 86 (69.9) | 0.863 | 0 |
| Diabetes *mellitus* – n. (%) | 96 (28.9) | 61 (29.2) | 35 (28.5) | 0.887 | 0 |
| COPD – n. (%) | 43 (13.0) | 19 (9.1) | 24 (19.5) | **0.006** | 0 |
| AFib – n. (%) | 139 (41.9) | 80 (38.3) | 59 (48.0) | 0.084 | 0 |
| **Heart Failure characterization** | | | | | |
| Ischemic etiology – n. (%) | 131 (39.5) | 81 (38.8) | 50 (40.7) | 0.733 | 0 |
| LVEF (%) | 41.9 ± 16.0 | 41.4 ± 14.9 | 42.8 ± 17.9 | 0.530 | 27.1 |
| LVEF <40% - n. (%) | 101 (30.4) | 70 (33.5) | 31 (25.2) | 0.113 |  |
| Beta-blockers – n. (%) | 158 (47.6) | 101 (48.3) | 57 (46.3) | 0.727 | 0 |
| ACEi/ARBs – n. (%) | 161 (48.5) | 106 (50.7) | 55 (44.7) | 0.291 | 0 |
| MRAs – n. (%) | 41 (12.3) | 24 (11.5) | 17 (13.8) | 0.532 | 0 |
| **Admission clinical assessment of congestion** | | | | | |
| Peripheral edema – n. (%) | 189 (56.9) | 119 (56.9) | 70 (56.9) | 0.996 | 0 |
| Pulmonary congestion* – n. (%) | 258 (77.7) | 160 (76.6) | 98 (79.7) | 0.509 | 0 |
| **Admission biochemical data** | | | | | |
| Urea (mg/dL) | 68.7 ± 43.7 | 65.0 ± 41.9 | 75.3 ± 46.1 | **0.040** | 3.0 |
| Creatinine (mg/dL) | 1.39 ± 0.69 | 1.34 ± 0.61 | 1.49 ± 0.81 | 0.060 | 3.3 |
| eGFR (ml/min/1.73m^2^) | 54.4 ± 29.8 | 54.8 ± 23.4 | 53.6 ± 38.8 | 0.742 | 2.1 |
| Sodium (mmol/L) | 136.8 ± 5.5 | 137.6 ± 4.9 | 135.4 ± 6.3 | **0.001** | 0.9 |
| Potassium (mmol/L) | 4.18 ± 0.75 | 4.17 ± 0.77 | 4.20 ± 0.73 | 0.750 | 1.2 |
| BNP/100 (pg/mL) | 10.8 (6.1 – 20.6) | 10.9 (6.1 – 20.0) | 10.5 (6.2 – 20.0) | 0.947 | 3.6 |
| Hemoglobin (g/dL) | 12.3 ± 2.0 | 12.7 ± 1.8 | 11.6 ± 2.0 | **<0.001** | 3.0 |
| RDW (%) | 14.5 ± 2.2 | 14.4 ± 2.2 | 14.8 ± 2.1 | 0.123 | 0 |
| RDW >15% - n. (%) | 110 (33.1) | 60 (28.7) | 50 (40.7) | **0.026** |  |
| **Discharge biochemical data** | | | | | |
| Creatinine (mg/dL) | 1.41 ± 0.81 | 1.34 ± 0.64 | 1.55 ± 1.03 | **0.047** | 23.1 |
| eGFR (ml/min/1.73m^2^) | 59.2 ± 34.5 | 59.1 ± 24.8 | 59.5 ± 47.6 | 0.926 | 23.1 |
| BNP/100 (pg/mL) | 4.6 (2.6 – 9.9) | 4.5 (2.7 – 9.2) | 11.1 (1.9 – 32.7) | 0.341 | 56.3 |
| RDW (%) | 14.6 ± 2.3 | 14.0 ± 2.1 | 15.5 ± 2.4 | **<0.001** | 0 |
| RDW >15% - n. (%) | 110 (33.1) | 47 (22.5) | 63 (51.2) | **<0.001** |  |
| ΔRDW (%) | 0.25 ± 5.50 | -2.61 ± 2.35 | 5.10 ± 5.93 | **<0.001** |  |
| **Length of Stay and Events** | | | | | |
| Length of stay (days) | 10 (7 – 15) | 10 (6 – 15) | 13 (6 - 14) | **0.002** | 0 |
| 180-day ACM – n. (%) | 38 (11.4) | 19 (9.1) | 19 (15.4) | 0.079 | 0 |

Legend: n., number; COPD, chronic obstructive pulmonary disease; AFib, atrial fibrillation; LVEF, left ventricular ejection fraction; ACEi/ARBs, angiotensin converting enzyme inhibitors/angiotensin receptor blockers; MRAs, mineralocorticoid receptor antagonists; BNP, brain natriuretic peptide; RDW, red blood cell distribution width; Δ, adjusted delta (discharge-admission/admission*100); ACM, all-cause mortality.

eGFR was calculated by the modified diet and renal disease (MDRD) formula.

**Bold**, significant value (p≤0.05).

*Includes patients with respiratory rate >25 cycles/min or SpO2 <90% or rales.

Supplemental Table 2. Multiple linear regression analysis of RDW at admission, discharge and ΔRDW (Paris cohort)

| **Admission RDW** | **Adjusted R^2^** | **Beta** | **Intercept B (95% CI)** | **Sd. Error** | **P-value** |
| --- | --- | --- | --- | --- | --- |
| Overall model fit | 0.15 | - | - | 1.96 | **<0.001** |
| Constant | - | - | 19.18 (16.89 to 21.47) | 1.16 | **<0.001** |
| Hemoglobin (g/dL) | 0.06 | -0.26 | -0.29 (-0.40 to -0.17) | 0.06 | **<0.001** |
| Atrial fibrillation (yes) | 0.03 | 0.23 | 0.98 (0.52 to 1.44) | 0.24 | **<0.001** |
| Peripheral edema (yes) | 0.02 | 0.11 | 0.45 (0.01 to 0.90) | 0.23 | **0.007** |
| BNP/100 (pg/mL) | 0.03 | 0.16 | 0.03 (0.01 to 0.04) | 0.01 | **0.002** |
| **Discharge RDW** | **Adjusted R^2^** | **Beta** | **Intercept B (95% CI)** | **Sd. Error** | **P-value** |
| Overall model fit | 0.88 | - | - | 0.81 | **<0.001** |
| Constant | - | - | 1.74 0.48 to 3.01) | 0.64 | **0.007** |
| Admission RDW (%) | 0.87 | 0.91 | 0.99 (0.95 to 1.03) | 0.02 | **<0.001** |
| Hemoglobin (g/dL) | 0.01 | -0.09 | -0.10 (-0.15 to -0.06) | 0.02 | **<0.001** |
| **ΔRDW** | **Adjusted R^2^** | **Beta** | **Intercept B (95% CI)** | **Sd. Error** | **P-value** |
| Overall model fit | 0.00 | - | - | 5.59 | 0.467 |

Bivariate correlations were first assessed, after which the variables with significant correlations were entered in the linear regression model in which a backward selection was used. Only the final variables retained in the model are presented.

In the admission RDW model, the following variables were tested: age; sex; atrial fibrillation (yes/no); LVEF; peripheral edema (yes/no); pulmonary congestion (yes/no); urea; creatinine; sodium; potassium; hemoglobin; BNP /100 (admission values were used unless stated otherwise).

In the discharge RDW model, the following variables were tested: age; sex; atrial fibrillation (yes/no); LVEF; peripheral edema (yes/no); pulmonary congestion (yes/no); urea; discharge creatinine; sodium; potassium; hemoglobin; discharge BNP /100; RDW (admission values were used unless stated otherwise).

In the ΔRDW model, the following variables were tested: the following variables were tested: age; sex; atrial fibrillation (yes/no); LVEF; peripheral edema (yes/no); pulmonary congestion (yes/no); urea; creatinine; sodium; potassium; hemoglobin; BNP /100; RDW (admission values were used unless stated otherwise).

**Bold**, significant value (p≤0.05).

Legend: Sd. Error, standard error; RDW, red blood cell distribution width; Δ, adjusted delta (discharge-admission/admission*100); BNP, brain natriuretic peptide.

Supplemental Table 3. Multiple logistic regression analysis of RDW (≤15 vs. >15% at admission and discharge), ΔRDW (≤0 vs. >0) (Paris cohort)

| **RDW>15% at admission** | **OR (95% CI)** | **P-value** |
| --- | --- | --- |
| Atrial fibrillation (yes) | 1.79 (0.98 - 3.26) | 0.059 |
| Hemoglobin (g/dL) | 0.79 (0.68 - 0.93) | **0.004** |
| **RDW>15% at discharge** | **OR (95% CI)** | **P-value** |
| Admission RDW (%) | 6.84 (4.35 - 10.78) | **<0.001** |
| **ΔRDW >0** | **OR (95% CI)** | **P-value** |
| Hemoglobin (g/dL) | 0.70 (0.59 – 0.82) | **<0.001** |

A backward model was used and only the final variables retained in the model are presented.

In the admission RDW model, the following variables were tested: age; sex; atrial fibrillation (yes/no); LVEF; peripheral edema (yes/no); pulmonary congestion (yes/no); urea; creatinine; sodium; potassium; hemoglobin; BNP /100 (admission values were used unless stated otherwise).

In the discharge RDW model, the following variables were tested: age; sex; atrial fibrillation (yes/no); LVEF; peripheral edema (yes/no); pulmonary congestion (yes/no); urea; discharge creatinine; sodium; potassium; hemoglobin; discharge BNP /100; RDW (admission values were used unless stated otherwise).

In the ΔRDW model, the following variables were tested: the following variables were tested: age; sex; atrial fibrillation (yes/no); LVEF; peripheral edema (yes/no); pulmonary congestion (yes/no); urea; creatinine; sodium; potassium; hemoglobin; BNP /100; RDW (admission values were used unless stated otherwise).

**Bold**, significant value (p≤0.05).

Legend: OR, odds ratio; RDW, red blood cell distribution width; Δ, adjusted delta (discharge-admission/admission).

Table 4. Univariable and multivariable Cox proportional hazards models for 180-day All-Cause Mortality (Paris cohort)

| **Variable** | **Univariable**  **HR (95% CI)** | **P-value** | **Model 1**  **HR (95% CI)** | **P-value** | **Model 2**  **HR (95% CI)** | **P-value** |
| --- | --- | --- | --- | --- | --- | --- |
| RDW (%) at admission | 1.10 (0.97 – 1.27) | 0.140 | 1.15 (1.00 – 1.32) | **0.047** | 1.13 (0.97 – 1.31) | 0.125 |
| RDW (%) at discharge | 1.16 (1.04 – 1.30) | **0.008** | 1.22 (1.08 – 1.37) | **0.001** | 1.22 (1.07 – 1.40) | **0.003** |
| RDW ≤15% at admission | Reference |  | Reference |  | Reference |  |
| RDW >15% at admission | 1.70 (0.88 – 3.27) | 0.114 | 1.21 (0.77 – 1.91) | 0.416 | 1.28 (0.79 – 2.07) | 0.312 |
| RDW ≤15% at discharge | Reference |  | Reference |  | Reference |  |
| RDW >15% at discharge | 2.53 (1.34 – 4.78) | **0.004** | 2.71 (1.43 – 5.14) | **0.002** | 2.77 (1.38 – 5.55) | **0.004** |
| ΔRDW | 1.09 (1.05 – 1.14) | **<0.001** | 1.09 (1.04 – 1.13) | **<0.001** | 1.09 (1.05 – 1.14) | **<0.001** |
| ΔRDW ≤0 | Reference |  | Reference |  | Reference |  |
| ΔRDW >0 | 1.82 (0.96 – 3.44) | 0.060 | 1.84 (0.97 – 3.47) | 0.062 | 2.03 (1.05 – 3.94) | **0.035** |
| RDW discharge ≤15% and ΔRDW ≤0 | Reference |  | Reference |  | Reference |  |
| RDW discharge ≤15% and ΔRDW >0 | 2.10 (0.84 – 5.21) | 0.111 | 2.01 (0.81 – 5.01) | 0.133 | 2.49 (0.98 – 6.31) | 0.055 |
| RDW discharge >15% and ΔRDW ≤0 | 3.41 (1.37 – 8.48) | **0.008** | 3.49 (1.40 – 8.71) | **0.007** | 3.81 (1.43 – 10.12) | **0.007** |
| RDW discharge >15% and ΔRDW >0 | 3.13 (1.36 – 7.24) | **0.007** | 3.38 (1.46 – 7.84) | **0.005** | 3.82 (1.57 – 9.32) | **0.003** |

RDW discharge ≤15% and ΔRDW ≤0 (n=162); RDW discharge ≤15% and ΔRDW >0 (n=60); RDW discharge >15 and ΔRDW ≤0 (n=47); RDW discharge >15 and ΔRDW >0 (n=63).

Model 1, adjusted for sex and age; Model 2, adjusted for sex, age, atrial fibrillation, admission hemoglobin and BNP.

No statistically significant interactions were found between the interest and explanatory variables.

**Bold**, significant value (p≤0.05).

Legend: HR, hazard ratio; RDW, red blood cell distribution width; Δ, adjusted delta (discharge-admission/admission*100); BNP, brain natriuretic peptide.

Supplemental Table 5. Net reclassification improvement and integrated discrimination improvement for predicting All-Cause Mortality at 180 days (Paris cohort)

| Added variable(s) | Baseline set of variables | NRI (%) | P-value | IDI (%) | P-value |
| --- | --- | --- | --- | --- | --- |
| Discharge RDW (continuous) | on top of age, LVEF, Hb, pCr and NT-pro BNP | 27.5 (-8.9 to 43.2) | 0.086 | 1.4 (-1.0 to 10.3) | 0.239 |
| Discharge RDW >15% |  | 25.2 (-2.1 to 40.8) | 0.073 | 2.5 (-0.2 to 11.6) | 0.126 |
| ΔRDW >0 |  | 18.2 (-7.2 to 33.2) | 0.106 | 2.2 (-0.5 to 8.2) | 0.139 |
| Discharge RDW >15% and ΔRDW >0 |  | 19.3 (3.4 to 42.7) | **0.020** | 3.5 (0.4 to 12.2) | **0.013** |

The prediction models include the following variables dichotomized according to the median: age in years (<78 vs. ≥78), left ventricular ejection fraction in % (<40 vs. ≥40), hemoglobin in g/dL (<12 vs. ≥ 12), creatinine in mg/dL (<1.3 vs. ≥ 1.3), BNP/100 in pg/mL(<11 vs. ≥11) at admission.

**Bold**, significant value (p≤0.05).

Legend: NRI, net reclassification improvement; IDI, integrated discrimination improvement; RDW, red blood cell distribution width; Δ, adjusted delta (discharge-admission/admission); NT-pro BNP, N-terminal pro brain natriuretic peptide; LVEF, left ventricular ejection fraction; Hb, hemoglobin, pCr, plasma creatinine

Table 6. Univariable and multivariable Cox proportional hazards models for 180-day All-Cause Mortality (Porto cohort)

| **Variable** | **Univariable**  **HR (95% CI)** | **P-value** | **Model 1**  **HR (95% CI)** | **P-value** | **Model 2**  **HR (95% CI)** | **P-value** |
| --- | --- | --- | --- | --- | --- | --- |
| RDW (%) at admission | 1.05 (0.96 – 1.14) | 0.279 | 1.05 (0.96 – 1.15) | 0.289 | 0.99 (0.88 – 1.12) | 0.884 |
| RDW (%) at discharge | 1.22 (1.09 – 1.36) | **0.001** | 1.21 (1.08 – 1.35) | **0.001** | 1.18 (1.03 – 1.36) | **0.020** |
| RDW ≤15% at admission | Reference |  | Reference |  | Reference |  |
| RDW >15% at admission | 1.54 (0.85 – 2.80) | 0.158 | 1.40 (0.76 – 2.57) | 0.280 | 1.03 (0.51 – 2.11) | 0.929 |
| RDW ≤15% at discharge | Reference |  | Reference |  | Reference |  |
| RDW >15% at discharge | 3.34 (1.77 – 6.28) | **<0.001** | 3.19 (1.67 – 6.11) | **<0.001** | 2.77 (1.33 – 5.76) | **0.007** |
| ΔRDW | 1.05 (1.01 – 1.09) | **0.012** | 1.05 (1.01 – 1.09) | **0.019** | 1.04 (1.00 – 1.08) | **0.042** |
| ΔRDW ≤0 | Reference |  | Reference |  | Reference |  |
| ΔRDW >0 | 2.78 (1.49 – 5.21) | **0.001** | 2.63 (1.39 – 4.98) | **0.003** | 2.40 (1.15 – 5.01) | **0.020** |
| RDW discharge ≤15% and ΔRDW ≤0 | Reference |  | Reference |  | Reference |  |
| RDW discharge ≤15% and ΔRDW >0 | 2.55 (0.95 – 6.84) | 0.064 | 2.48 (0.92 – 6.76) | 0.074 | 1.47 (0.46 – 4.75) | 0.513 |
| RDW discharge >15 and ΔRDW ≤0 | 3.20 (1.24 – 8.30) | **0.017** | 3.14 (1.19 – 8.28) | **0.021** | 1.77 (0.54 – 5.74) | 0.344 |
| RDW discharge >15 and ΔRDW >0 | 5.65 (2.49 – 12.80) | **<0.001** | 5.39 (2.33 – 12.45) | **<0.001** | 4.42 (1.76 – 11.08) | **0.002** |
| ΔRDW ≤0 and ΔHemoglobin >0 | Reference |  | Reference |  | Reference |  |
| ΔRDW ≤0 and ΔHemoglobin ≤0 | 0.67 (0.26 – 1.77) | 0.422 | 0.68 (0.26 – 1.81) | 0.225 | 1.48 (0.46 – 4.75) | 0..513 |
| ΔRDW >0 and ΔHemoglobin >0 | 2.31 (0.73 – 7.27) | 0.241 | 2.04 (0.64 – 6.52) | 0.229 | 1.77 (0.54 – 5.74) | 0.344 |
| ΔRDW >0 and ΔHemoglobin ≤0 | 2.10 (0.88 – 5.03) | 0.096 | 2.06 (0.86 – 4.95) | 0.104 | 4.42 (1.76 – 11.02) | **0.002** |

RDW discharge ≤15% and ΔRDW ≤0 (n=77); RDW discharge ≤15% and ΔRDW >0 (n=26); RDW discharge >15 and ΔRDW ≤0 (n=26); RDW discharge >15 and ΔRDW >0 (n=32).

ΔRDW ≤0 and ΔHemoglobin >0 (n=34); ΔRDW ≤0 and ΔHemoglobin ≤0 (n=68); ΔRDW >0 and ΔHemoglobin >0 (n=11); ΔRDW >0 and ΔHemoglobin ≤0 (n=47).

ΔHemoglobin >0 = hemoconcentration *i.e*. increase in hemoglobin from admission to discharge, and

ΔHemoglobin ≤0 = no hemoconcentration *i.e*. decrease or no increase in hemoglobin from admission to discharge

Model 1, adjusted for sex and age; Model 2, adjusted for sex, age, atrial fibrillation, admission hemoglobin and NT-pro BNP.

No statistically significant interactions were found between the interest and explanatory variables.

**Bold**, significant value (p≤0.05).

Legend: HR, hazard ratio; RDW, red blood cell distribution width; Δ, adjusted delta (discharge-admission/admission*100); NT-pro BNP, N-terminal pro brain natriuretic peptide.

Table 7. Net reclassification improvement and integrated discrimination improvement for predicting All-Cause Mortality at 180 days (Porto cohort)

| Added variable(s) | Baseline set of variables | NRI (%) | P-value | IDI (%) | P-value |
| --- | --- | --- | --- | --- | --- |
| Discharge RDW (continuous) | on top of age, LVEF, Hb, pCr and NT-pro BNP | 34.7 (-2.0 to 50.0) | 0.073 | 8.4 (1.1 to 19.6) | **0.020** |
| Discharge RDW >15% |  | 35.6 (3.6 to 55.1) | **0.040** | 7.5 (1.0 to 20.6) | **0.007** |
| ΔRDW >0 |  | 37.2 (6.0 to 55.4) | **0.020** | 7.4 (1.0 to 19.0) | **0.013** |
| Discharge RDW >15% and ΔRDW >0 |  | 31.9 (17.6 to 47.1) | **<0.001** | 12.6 (3.5 to 27.3) | **0.007** |
| ΔHemoglobin >0 (hemoconcentration) |  | 2.7 (-9.3 to 23.0) | 1.000 | 0.1 (-0.2 to 4.5) | 0.292 |

The prediction models include the following variables dichotomized according to the median: age in years (<77 vs. ≥77), left ventricular ejection fraction in % (<45 vs. ≥45), hemoglobin in g/dL (<12 vs. ≥ 12), creatinine in mg/dL (<1.5 vs. ≥ 1.5), NT-pro BNP in pg/mL(<3500 vs. ≥ 3500) at admission.

ΔHemoglobin >0 = hemoconcentration *i.e*. increase in hemoglobin from admission to discharge.

**Bold**, significant value (p≤0.05).

Legend: NRI, net reclassification improvement; IDI, integrated discrimination improvement; RDW, red blood cell distribution width; Δ, adjusted delta (discharge-amission/admission*100).
